# Urban–rural disparities in maternal healthcare knowledge, attitudes, and practices among women with a recent live birth in Abuja and Gombe states, Nigeria

**DOI:** 10.3389/fpubh.2026.1909190

**Published:** 2026-08-26

**Authors:** Chinelo Lynda Okoye, Precious Ojo Uahomo, Chinasa Godsgift Eni, Korfii Uebari, Chidi Emmanuel Ezerioha, Sussana Chinemerem Oguaba, Naomi Akpevwe Mogborukor, Umar Abubakar, Asemebo Goodness Okorosa, Oluwaseun Fadeyi

**Affiliations:** 1 Department of Health Promotion and Community Health, Faculty of Health Sciences, American University of Beirut, Beirut, Lebanon; 2 Department of Pharmacology and Therapeutics, Faculty of Basic Clinical Sciences, University of Abuja, Gwagwalada, Nigeria; 3 School of Public Health, University of Port Harcourt, Nigeria; 4 Department of Pharmacology, Faculty of Basic Medical Sciences, Southern Delta University, Ozoro, Nigeria; 5 Department of Human Nutrition and Dietetics, University of Calabar, Calabar, Nigeria; 6 Department of Experimental Pharmacology and Toxicology, Faculty of Pharmaceutical Sciences, University of Port Harcourt, Choba, Nigeria; 7 Department of Public Health, Faculty of Basic and Applied Biological Sciences, Ahmadu Bello University, Zaria, Nigeria; 8 Tropical Institute of Public Health, Abuja, Nigeria; 9 Centre for Environmental Management, University of the Free State, Bloemfontein, South Africa

**Keywords:** attitudes, cross-sectional study, knowledge, maternal health, Nigeria, practices, socioeconomic factors, urban–rural disparities

## Abstract

**Background:**

Nigeria bears the highest burden of maternal mortality globally, with marked urban–rural disparities. Rural women have significantly lower uptake of skilled antenatal care (60.7% vs. 91.8%) and facility-based delivery (48.5% vs. 81.3%). However, comparative data on maternal knowledge, attitudes, and practices (KAP) remain limited in northern Nigeria. This study compared maternal healthcare KAP between urban and rural women and identified predictors of good outcomes.

**Methods:**

A community-based cross-sectional study was conducted in Abuja (urban) and Gombe State (rural) from June to July 2025. Women aged 15–49 years with a live birth in the preceding 12 months were included. Multi-stage stratified cluster sampling selected 1,218 women, yielding a final analytical sample of 1,045 (606 urban, 439 rural). A pre-tested interviewer-administered questionnaire assessed maternal knowledge (15 items, *α* = 0.84), attitudes (11 Likert items, *α* = 0.88), and practices (14 items, *α* = 0.81). Chi-square tests compared urban–rural differences. Hierarchical multivariable logistic regression, guided by the Social Ecological Model, identified predictors of good knowledge, positive attitude, and good practice, with variables entered in blocks according to SEM levels.

**Results:**

Urban women had significantly higher proportions of good knowledge (66.3% vs. 25.5%, *p* < 0.001), positive attitudes (87.1% vs. 51.9%, *p* < 0.001), and good practices (57.8% vs. 23.9%, *p* < 0.001). Urban residence independently predicted good knowledge (AOR = 3.84, 95% CI: 2.67–5.53), positive attitude (AOR = 3.41, 95% CI: 2.38–4.89), and good practice (AOR = 2.84, 95% CI: 1.97–4.09). Secondary/tertiary education (AORs: 3.16, 2.11, 1.88) and higher income (AORs: 1.72, 1.54, 1.57) were consistent predictors. Good knowledge strongly predicted positive attitude (AOR = 4.28) and good practice (AOR = 3.91). Perceived affordability (knowledge AOR = 1.68; practice AOR = 1.81) and feeling safe at health facilities (knowledge AOR = 1.52; attitude AOR = 1.72) were significant predictors; respectful healthcare workers predicted attitude (AOR = 1.97) and practice (AOR = 1.69).

**Conclusion:**

Substantial urban–rural gaps in maternal KAP persist, driven by education, income, and health system factors. Interventions that promote girls’ education, deliver targeted community health education, improve affordability and safety of maternal services, and strengthen respectful maternity care may reduce disparities in rural Nigeria.

## 1 Introduction

Maternal mortality, a key indicator of health system performance and access to quality maternal healthcare services, has declined globally (1). However, despite a 40% reduction from 2000 to 2023 (328 to 197 deaths per 100,000 live births), maternal death still occurs every 2 min, accounting for approximately 700 deaths daily worldwide (1). These outcomes remain unequally distributed, with a global maternal mortality ratio of 292 among rural women compared to 100 among urban women, based on a 2022 simulation-based analysis (2), reflecting persistent inequities in access to and utilization of maternal healthcare services.

In 2023, Sub-Saharan Africa, including Nigeria, accounted for 70% of global maternal deaths, with Nigeria alone contributing 28% (1). Nigeria currently records one of the highest maternal mortality ratios globally, with marked regional disparities. According to the Nigeria Demographic and Health Survey 2023--24, maternal mortality is approximately five times higher in the northern region compared to the south, with rural areas bearing a disproportionate burden (3). Within northern Nigeria, estimates suggest a maternal mortality ratio of 828 per 100,000 live births in rural areas compared to 351 per 100,000 in urban areas (4).

These disparities are largely driven by inequalities in access to quality maternal healthcare services, including antenatal care, skilled birth attendance, and postnatal care (1). Evidence from the Nigeria Demographic and Health Survey shows consistently lower uptake of these services among rural women, despite higher fertility and birth rates compared to their urban counterparts (3).

Specifically, marked disparities in maternal healthcare utilization were evident between predominantly urban regions, such as the Federal Capital Territory (FCT), and predominantly rural regions, such as Gombe State, among women who had a live birth or stillbirth within 2 years of the NDHS (3). Although Gombe recorded more women with recent births (208 vs. 149), women in FCT consistently reported higher maternal healthcare utilization. Skilled antenatal care attendance was 91.8% in FCT compared to 60.7% in Gombe, while facility-based delivery was 81.3% versus 48.5%, and postnatal care uptake was 82.3% versus 44.9%, respectively (3). These findings reflect a consistent and substantial urban–rural gradient in maternal healthcare utilization.

Key determinants of these disparities include structural, systemic, and individual-level factors such as distance to healthcare facilities, poverty, limited knowledge about maternal healthcare, restricted access to education and health information, and shortage of skilled health workers (5, 6). These factors collectively limit women’s ability to access and use essential maternal health services, particularly in rural settings.

Among these determinants, access to education and health information plays a major role in shaping women’s knowledge, attitudes, and practices (KAP) regarding maternal healthcare, which are associated with service utilization (7, 8). Maternal healthcare knowledge has been shown to be associated with improved awareness of pregnancy danger signs, antenatal care importance, skilled delivery, and postnatal care, thereby supporting timely healthcare seeking (9). Studies also suggest that improved knowledge is associated with more positive attitudes toward maternal healthcare services, which may enhance utilization (7, 8). However, attitudes are also influenced by prior healthcare experiences and perceptions of provider competence, highlighting that both health system and individual factors shape maternal healthcare behaviour (6).

Furthermore, knowledge and attitudes have been associated with improved maternal healthcare practices, defined as the adoption of recommended maternal health behaviours during pregnancy, delivery, and the postpartum period, including utilization of maternal healthcare services, adherence to prescribed medications, and uptake of at least three doses of intermittent preventive treatment in pregnancy (IPTp3+) (7, 10).

Despite growing evidence on KAP and maternal healthcare utilization, existing studies have largely focused on single communities or individual states (11–13). A few studies have compared urban–rural differences in specific maternal health outcomes within individual states, such as birth preparedness in Enugu State (14). However, to our knowledge, no published study has directly compared maternal healthcare KAP between an urban and a rural population using contrasting settings as distinct as the Federal Capital Territory (predominantly urban) and Gombe State (predominantly rural) in northern Nigeria. Studies that have compared urban and rural populations have typically focused on single states or on service utilization alone rather than the full KAP continuum. This gap is particularly relevant given that Abuja and Gombe differ markedly in urbanization, socioeconomic conditions, and healthcare access. Understanding these differences may provide evidence for targeted interventions to reduce inequities in maternal healthcare utilization.

### 1.1 Conceptual framework

This study was guided by the Social Ecological Model (SEM), which posits that health behaviours are shaped by the interaction of factors operating at the individual, interpersonal, institutional, community, and policy levels (15). Within this framework, maternal healthcare knowledge, attitudes, and practices are influenced by personal characteristics, social relationships, healthcare system attributes, and broader structural determinants that collectively determine health-seeking behaviour and service utilization.

At the individual level, factors such as age, educational attainment, household income, and maternal healthcare knowledge influence health literacy, risk perception, and self-efficacy. The interpersonal level encompasses family support and household decision-making dynamics that affect women’s autonomy in seeking maternal healthcare. At the institutional level, characteristics of the healthcare system, including service affordability, respectful treatment by healthcare providers, and perceptions of safety within health facilities, influence healthcare utilization and patient experiences. The community level reflects contextual factors such as place of residence, prevailing social norms, and access to health information, while the policy level captures broader structural influences, including health system financing, resource allocation, and policies that determine the availability and accessibility of maternal health services.

The SEM informed the selection of explanatory variables in this study, with education, household income, place of residence, perceived affordability of services, respectful maternity care, and perceived safety representing different ecological levels. Furthermore, the study incorporated the Knowledge-Attitude-Practice (KAP) model, which proposes that knowledge influences attitudes, and attitudes subsequently shape health-related practices (16). The KAP model provides a useful heuristic for understanding the sequential pathway through which health education and information may influence behaviour.

**Figure 1:**
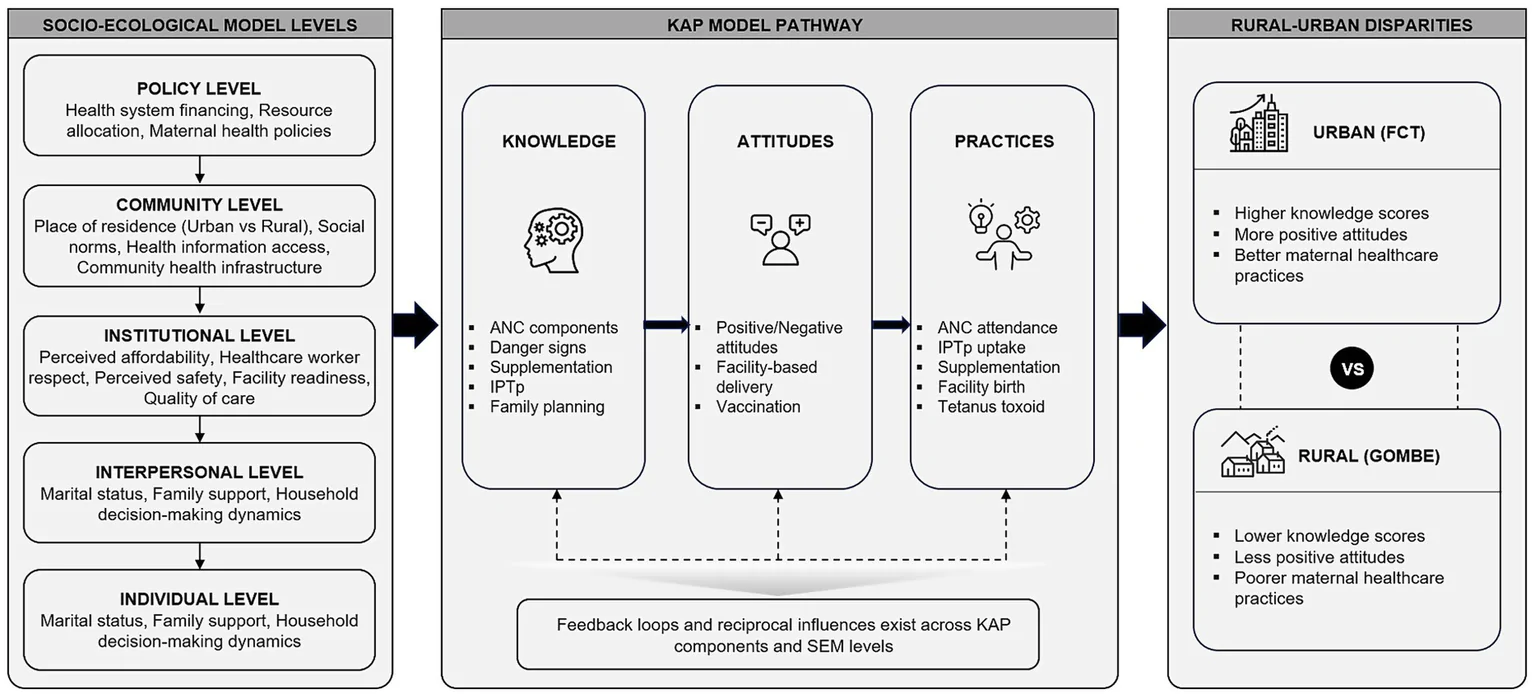
Integrated SEM-KAP conceptual framework for urban–rural disparities in maternal healthcare.

Figure 1 presents the integrated SEM-KAP framework guiding this study. The framework illustrates how factors at multiple ecological levels (individual, interpersonal, institutional, community, and policy) influence maternal healthcare knowledge, which in turn shapes attitudes and ultimately practices. The framework acknowledges bidirectional influences, particularly between institutional factors and attitudes, recognising that healthcare experiences can shape attitudes independently of knowledge.

### 1.2 Study objectives

This study therefore had two objectives: first, to conduct a comparative assessment of maternal healthcare knowledge, attitudes, and practices among urban mothers in the Federal Capital Territory and rural mothers in Gombe State, guided by the Social Ecological Model; and second, to identify factors associated with good knowledge, positive attitudes, and good practice in these populations, with the aim of informing multi-level interventions to reduce urban–rural disparities.

## 2 Methods

### 2.1 Study design

This was a community-based comparative cross-sectional study designed to assess KAP related to maternal healthcare (antenatal and intrapartum care) and to compare these between urban and rural women in Abuja and Gombe States, Nigeria. The “comparative” aspect refers to the explicit comparison of outcomes between two geographically and socio-demographically distinct populations (urban FCT and rural Gombe). The cross-sectional design captured data at a single point in time, providing a snapshot of maternal healthcare KAP and allowing for the examination of associations between predictor variables and outcomes. The study also examined factors associated with optimal utilization of maternal health services.

### 2.2 Study setting and urban–rural classification

The study was conducted in two geographically and socio-demographically distinct areas of Nigeria: the Federal Capital Territory (FCT, Abuja) and Gombe State. Abuja is Nigeria’s capital city, with relatively better health infrastructure, higher literacy rates, and greater access to maternal health services. Gombe State, located in north-eastern Nigeria, is predominantly rural with lower health indicator performance, including higher maternal mortality and lower skilled birth attendance (17).

To avoid ecological fallacy and misclassification bias, urban and rural status was not assigned at the state level. Instead, using the National Population Commission (NPC) classification from the 2018 census frame, we selected enumeration areas (EAs) classified as urban within the FCT (Abuja) and EAs classified as rural within Gombe State. The NPC classifies enumeration areas as urban based on criteria including population density (≥400 persons per square kilometre), availability of infrastructure (piped water, electricity, paved roads), and proportion of the population engaged in non-agricultural occupations (≥75%). Conversely, rural EAs are those that do not meet these criteria. This classification system was applied consistently across both study states. Only EAs that met the NPC’s official urban–rural designation were included. This ensured that the comparison was between genuinely urban and genuinely rural populations rather than between states. All 35 EAs selected from the FCT were urban, and all 35 EAs selected from Gombe State were rural according to NPC criteria (17).

### 2.3 Study population and eligibility

The target population was women of reproductive age (15–49 years) who had delivered a child within the 12 months preceding the survey, a recall period chosen to minimise recall bias while capturing recent maternal healthcare experiences, consistent with Demographic and Health Survey (DHS) methodology (18). The 12-month recall period was chosen to balance the competing demands of capturing recent maternal healthcare experiences while ensuring a sufficient sample size. This recall period is consistent with DHS methodology for maternal health indicators, which uses a 5-year recall for births but focuses on the most recent birth within 2–5 years (18). For service-specific indicators, the DHS uses a 12-month recall period for ANC content and postnatal care (17). Women were included if they were aged 15–49 years, had a live birth in the 12 months preceding the survey, were resident in the selected enumeration areas for at least 6 months, and provided voluntary informed consent (with assent for minors aged 15–17 years obtained alongside parental or guardian consent). Women were excluded if they were unable to communicate due to severe illness or cognitive impairment, or if they refused to participate.

### 2.4 Sampling procedure

A multi-stage stratified cluster sampling design was used. In stage one (selection of clusters), EAs from the 2018 National Population Commission census frame were used as primary sampling units in each study area (Abuja and Gombe), with EAs stratified into urban (Abuja) and rural (Gombe) strata and 35 EAs randomly selected from each stratum using probability proportional to population size (PPS). In stage two (selection of households), a systematic random sampling of households was performed within each selected EA; field workers conducted a household listing, and households were selected with a sampling interval varying by EA population density to achieve the required number of eligible women. In stage three (selection of respondents), one woman was randomly selected using a simple random method (lottery) from each selected household with at least one eligible woman when more than one was present; households with no eligible woman were skipped and replaced by the next household on the list. Sampling weights were calculated as the inverse of the probability of selection at each stage and were applied in all analyses to account for unequal selection probabilities and non-response.

### 2.5 Sample size determination

The sample size was calculated prospectively before data collection. Because the study had multiple outcomes (knowledge, attitude, practice) rather than a single hypothesis test, the sample size was calculated using the precision-based (estimation) approach for a proportion formula (for large population):


$$n=DEFF\times \frac{Z^{2}\times p\left(1-p\right)}{d^{2}}$$

Based on a conservative expected proportion of good maternal healthcare practice of 50% (assumed in the absence of a reliable prior estimate from comparable Nigerian studies using a composite practice score), a desired absolute precision of ±4%, a 95% confidence level (*Z* = 1.96), a design effect of 1.8 to account for cluster sampling based on the average DEFF for maternal health indicators from the Nigeria DHS 2018 (17, 19), and a 10% non-response rate, the minimum required total sample size was calculated as 1,200, or approximately 600 per stratum (urban and rural). The target sample size was therefore 1,200 (600 urban, 600 rural). The final achieved sample was 1,218, which reduced to 1,045 after data cleaning (606 urban, 439 rural), with the lower rural number reflecting higher non-response due to seasonal migration and security constraints in parts of Gombe State. With the achieved sample of 1,045 (606 urban, 439 rural) and a two-sided alpha of 0.05, the study had 99% power to detect a 20%-point difference in good practice between urban and rural groups (assuming 50% urban and 30% rural) and 95% power to detect a 15%-point difference, indicating that the sample was adequately powered for the primary comparisons.

### 2.6 Data collection tools

A structured, interviewer-administered questionnaire was developed following a review of literature and adaptation of validated KAP instruments (20–22). The questionnaire was originally prepared in English, then translated into Hausa and Pidgin English, and back-translated by independent translators to ensure semantic equivalence. The questionnaire covered the following domains: sociodemographic characteristics (8 items), maternal health knowledge (15 items), attitudes toward maternal health (11 Likert-scale items), and maternal healthcare practices (14 items).

Content validity was assessed by a panel of five public health experts (three maternal health specialists and two epidemiologists) who independently evaluated each item for relevance, clarity, and comprehensiveness. The panel rated each item on a 4-point relevance scale (1 = not relevant to 4 = highly relevant). The Item-level Content Validity Index (I-CVI) was calculated as the proportion of experts rating the item as 3 or 4, with I-CVI ≥ 0.78 considered acceptable. The Scale-level Content Validity Index (S-CVI), calculated using the universal agreement method (S-CVI/UA), was 0.89, and using the average method (S-CVI/Ave), was 0.92, both exceeding the recommended threshold of 0.80 (23). The panel also assessed clarity and comprehensiveness; ambiguous items were reworded, and no items were deleted as all met the I-CVI threshold. The final questionnaire had an average administration time of 30–35 min based on pre-testing with 50 women (25 urban, 25 rural) from communities not included in the final sample. Based on pre-test results, ambiguous questions were reworded, and the average administration time was confirmed as 30–35 min. The pre-test data were not included in the main analysis. Internal consistency reliability (Cronbach’s alpha) calculated from the main study data was 0.84 for the maternal healthcare knowledge scale, 0.88 for the attitude scale, and 0.81 for the practice scale, indicating good to excellent internal consistency across all domains.

### 2.7 Data collection procedure

Twenty trained female enumerators (10 per state) with prior experience in maternal health surveys conducted face-to-face interviews at respondents’ homes in a private location (inside the home or a shaded outdoor area). Training lasted 3 days and covered ethical conduct, interview techniques, cultural sensitivity, questionnaire administration, and referral of women with danger signs. Field supervisors (four per state) and a central coordinator (study lead) oversaw data collection. Each completed questionnaire was checked daily for completeness and consistency. Spot checks were performed on 10% of households by supervisors to validate responses (re-interviewing 5% of respondents). Data were collected over 4 weeks (June–July 2025) to avoid the rainy season, which can limit access to rural areas. Oral and written informed consent were obtained from each participant before the interview. For adolescents aged 15–17 years, parental/guardian consent was obtained in addition to adolescent assent.

### 2.8 Variables and scoring

#### 2.8.1 Outcome variables

- *Maternal healthcare knowledge*: Maternal healthcare knowledge was assessed using 15 knowledge items covering awareness and understanding of: recommended number of ANC visits, key danger signs during pregnancy, appropriate place for ANC, importance of laboratory investigations, tetanus toxoid vaccination (recommended doses), IPTp-SP, folic acid/iron/calcium supplementation, additional food needs during pregnancy, family planning before pregnancy, and the effects of alcohol and smoking on pregnancy outcomes. For each item, correct responses were scored as 1, while incorrect responses were scored as 0. “Do not know” responses were also scored as 0 (incorrect), consistent with standard KAP scoring practice. This conservative approach ensures that only women with demonstrable knowledge are classified as having good knowledge. For conditional knowledge items (e.g., identification of specific danger signs among women who reported knowing any danger signs), respondents who answered “No” to the screening question (“Do you know any danger signs during pregnancy?”) were automatically scored as 0 for the corresponding follow-up items. The total possible score range was 0 to 15. Knowledge scores were categorized into three levels: poor knowledge [0–5], moderate knowledge [6–10], and good knowledge [11–15]. The scale demonstrated good internal consistency (Cronbach’s alpha = 0.84).
- *Maternal healthcare attitude*: Maternal healthcare attitude was assessed using 11 Likert-scale statements covering attitudes toward: the importance of regular ANC visits, the need for pregnant women to know danger signs, the value of laboratory investigations during pregnancy, necessity of tetanus toxoid vaccination, effectiveness of IPTp-SP for malaria prevention, importance of iron and folic acid supplementation, need for additional nutritious food during pregnancy, preference for facility-based delivery, the view that traditional healers should not replace hospital care, the harmfulness of smoking and alcohol during pregnancy, and the importance of family planning counselling. Responses were scored as follows: Strongly Agree = 5, Agree = 4, Neutral = 3, Disagree = 2, and Strongly Disagree = 1. All 11 items were summed to generate a total attitude score ranging from 11 to 55. Higher scores indicated more positive attitudes toward maternal healthcare. For attitude dichotomisation, we used a pre-specified *a priori* cut-off based on 80% of the maximum score, as recommended for Likert-scale KAP studies (23, 24). This approach is also consistent with standard practice in health survey methodology, where an 80% threshold is commonly used to define ‘positive’ or ‘good’ outcomes (25). The maximum possible score was 55; 80% of this is 44. Therefore, positive attitude was defined as a score of ≥44, and negative attitude as <44. This approach avoids the sample-dependency of median splits and enhances comparability across studies. The mean attitude score was 46.8 (SD = 6.2) among urban women and 38.5 (SD = 8.4) among rural women, representing a statistically significant difference (*p* < 0.001). The scale demonstrated excellent internal consistency (Cronbach’s alpha = 0.88).
- *Maternal healthcare practice*: Maternal healthcare practice was assessed using 14 practice items covering: ANC attendance during last pregnancy, timing of first ANC visit (trimester), number of ANC visits attended (categorised as <4, 4–7, or ≥8 visits), place of ANC receipt (government hospital, primary health centre, private hospital, traditional birth home), experience of danger signs during pregnancy, receipt of tetanus toxoid vaccination and number of doses received (1, 2, or ≥3 doses), receipt of IPTp-SP and number of doses received (1, 2, or ≥3 doses), use of folic acid supplementation, use of iron and calcium supplements, use of family planning methods before pregnancy, and consumption of additional meals during pregnancy. For each item, appropriate practice was scored as 1 and inappropriate practice as 0, following national and WHO guidelines (21). For conditional practice items (e.g., number of ANC visits, tetanus toxoid doses, IPTp-SP doses), respondents who answered “No” to the screening question (e.g., “Did you attend ANC during your last pregnancy?”) were automatically scored as 0 for all related follow-up items. This conservative approach ensured that non-utilization was not obscured by conditional questions. Total practice scores ranged from 0 to 14 and were categorised into three levels: poor practice [0–5], fair practice [6–9], and good practice [10–14]. The scale demonstrated acceptable internal consistency (Cronbach’s alpha = 0.81).

#### 2.8.2 Independent variables

As guided by the SEM and the KAP model, the selection of independent variables was theoretically driven. The SEM framework posits that health behaviours are influenced by multiple, interacting levels: individual, interpersonal, community, institutional, and policy factors (15). The independent variables were categorised as follows:

- *Individual-level variables*: Individual-level variables included age group (<30 years vs. ≥30 years), highest level of education (none/primary vs. secondary/tertiary), employment status (employed/self-employed vs. unemployed), monthly household income (<₦50,000 vs. ≥₦50,000), and parity (1 child, 2–4 children, or ≥5 children). The monthly household income cutoff of ₦50,000 was selected based on the Nigerian national minimum wage (₦30,000 per month) and the World Bank’s Nigeria poverty benchmark. At the time of data collection, the national minimum wage was ₦30,000; the ₦50,000 cutoff was chosen to distinguish households earning at or near the minimum wage (below ₦50,000) from those with incomes substantially above the minimum wage (≥₦50,000). This cutoff also approximates the median household income in our sample and is consistent with thresholds used in previous Nigerian maternal health studies (26).
- *Interpersonal- and Institutional-level variables*: Interpersonal-level variables included marital status (married vs. others: single, separated, divorced, or widowed). Institutional-level variables comprised perceived affordability of maternal healthcare services, healthcare worker respect during ANC visits, and feeling safe while attending health facilities, each categorized as yes or no.
- *Community-level variables*: The community-level variable was place of residence (urban vs. rural), classified according to the NPC EA classification. In addition, good maternal healthcare knowledge was included as a predictor in the attitude and practice regression models, while positive maternal healthcare attitude was included as a predictor in the practice regression model.

This multi-level approach allowed us to examine how factors at different ecological levels independently and collectively predict maternal healthcare KAP. Table 1 presents a summary of all variables, their measurement methods, and scoring criteria.

**Table 1:** Summary of variables, measurement methods, and scoring criteria.

Variable domain	Category	Variable	Measurement method	Scoring/categorization
Outcome variables	Maternal healthcare knowledge	15 knowledge items	Interviewer-administered questionnaire	Correct = 1, Incorrect = 0; Total 0–15; Poor (0–5), Moderate (6–10), Good (11–15); *α* = 0.84
	Maternal healthcare attitude	11 Likert-scale statements	Interviewer-administered questionnaire	Strongly Agree = 5 to Strongly Disagree = 1; Total 11–55; Positive (≥44), Negative (<44); *α* = 0.88
	Maternal healthcare practice	14 practice items	Interviewer-administered questionnaire	Appropriate = 1, Inappropriate = 0; Total 0–14; Poor (0–5), Fair (6–9), Good (10–14); *α* = 0.81
Independent variables				
Individual-level	Age	Self-report	Categorical	<30 years vs. ≥30 years
	Education	Self-report	Categorical	None/Primary vs. Secondary/Tertiary
	Employment	Self-report	Categorical	Employed/Self-employed vs. Unemployed
	Monthly household income	Self-report	Categorical	<₦50,000 vs. ≥₦50,000
	Parity	Self-report	Categorical	1 child, 2–4 children, ≥5 children
Interpersonal-level	Marital status	Self-report	Categorical	Married vs. Others
Institutional-level	Perceived affordability	Self-report (Yes/No)	Binary	Yes vs. No
	Healthcare worker respect	Self-report (Yes/No)	Binary	Yes vs. No
	Perceived safety	Self-report (Yes/No)	Binary	Yes vs. No
Community-level	Place of residence	NPC classification	Binary	Urban vs. Rural
Additional Predictors	Good knowledge	From knowledge score	Binary	Yes vs. No (in attitude and practice models)
	Positive attitude	From attitude score	Binary	Yes vs. No (in practice model)

### 2.9 Statistical analysis

Data were entered into EpiData version 3.1 with double-entry verification and analysed using IBM SPSS version 26 and Stata version 17.0 (for complex survey analysis), with sampling weights applied in all analyses to account for the multi-stage cluster design. The design effect for the primary outcome (good practice) was 1.52, and the effective sample size was approximately 687.

Descriptive analysis involved frequencies and percentages for categorical variables, while comparisons between urban and rural respondents used chi-square tests to assess differences in proportions. Bivariate analysis was conducted to examine associations between each independent variable and the primary outcomes (good maternal knowledge, positive maternal attitude, and good maternal practice) using chi-square tests, with a *p*-value <0.05 considered statistically significant.

Variables for multivariable logistic regression were selected *a priori* based on literature review, subject matter knowledge, and the Social Ecological Model framework, not based solely on bivariate significance. All variables identified through the conceptual framework were included in the regression models regardless of their bivariate significance. This approach ensures that the models are theoretically grounded and avoids the problem of data-driven variable selection, which can lead to overfitting and spurious associations. However, parity and marital status, though included in bivariate analyses, were not included in multivariable models due to collinearity with age and residence, respectively.

Variables were entered hierarchically based on the Social Ecological Model framework, with blocks of variables entered sequentially to examine the independent contribution of each SEM level. Block 1 (individual-level) included age group, education, employment, income, and parity. Block 2 (interpersonal-level) included marital status. Block 3 (institutional-level) included perceived affordability, healthcare worker respect, and safety perception. Block 4 (community-level) included place of residence. Good knowledge was added to the attitude model, and both good knowledge and positive attitude were added to the practice model, consistent with the KAP model’s hypothesised pathway. In the knowledge regression model, ANC attendance was not included as a predictor to avoid theoretical circularity, as ANC attendance is itself a practice outcome within the KAP framework. The hierarchical approach allowed us to quantify the variance explained by each SEM level (Nagelkerke R^2^) and assess the independent contribution of community-level factors after controlling for lower-level factors.

All multivariable logistic regression models were adjusted for the following variables: place of residence (community level), age group, education, employment, income (individual level), perceived affordability, healthcare worker respect, and safety perception (institutional level). The attitude model was additionally adjusted for good maternal healthcare knowledge, while the practice model was additionally adjusted for both good knowledge and positive attitude. Marital status (interpersonal level) and parity (individual level) were included in bivariate analyses but not in multivariable models due to collinearity with age and residence.

For the practice outcome, we used binary logistic regression (good practice vs. not good practice) rather than ordinal logistic regression for the following reasons: First, the primary research question focused on identifying factors associated with ‘good practice’ (the highest category) versus ‘not good practice’ (poor/fair combined), which represents a clinically meaningful dichotomy aligned with our objective of identifying predictors of optimal maternal healthcare practices. Second, the proportional odds assumption required for ordinal logistic regression was violated (test of parallel lines, *p* < 0.05), making binary logistic regression more appropriate. Third, binary logistic regression provides more straightforward interpretation of odds ratios for the outcome of interest.

Multicollinearity was assessed using variance inflation factor (VIF), and all variables had VIF < 3, indicating no problematic collinearity. Adjusted odds ratios (AOR) with 95% confidence intervals were reported. Model fit was evaluated using the area under the receiver operating characteristic (ROC) curve (AUC). The final models showed acceptable discrimination: knowledge model AUC = 0.78 (95% CI: 0.75–0.81), attitude model AUC = 0.82 (95% CI: 0.79–0.85), and practice model AUC = 0.79 (95% CI: 0.76–0.82). The Hosmer-Lemeshow goodness-of-fit test, while reported, is known to be sample-sensitive; we therefore relied primarily on AUC for model performance assessment.

Reliability analysis calculated Cronbach’s alpha (≥0.70 acceptable, ≥0.80 good) for each scale from the main study data: maternal knowledge *α* = 0.84, maternal attitude *α* = 0.88, maternal practice *α* = 0.81.

Missing data accounted for less than 3% of responses across all items. Little’s MCAR test indicated that missingness was completely at random (*χ*^2^ = 42.3, df = 38, *p* = 0.29). Cases with missing data on any variable included in a given analysis were excluded listwise from that analysis. The final analytical sample sizes were: knowledge model (*n* = 1,045), attitude model (*n* = 1,045), and practice model (*n* = 1,045). No variable had more than 2.8% missingness.

## 3 Results

### 3.1 Participant flow and response rate

**Figure 2:**
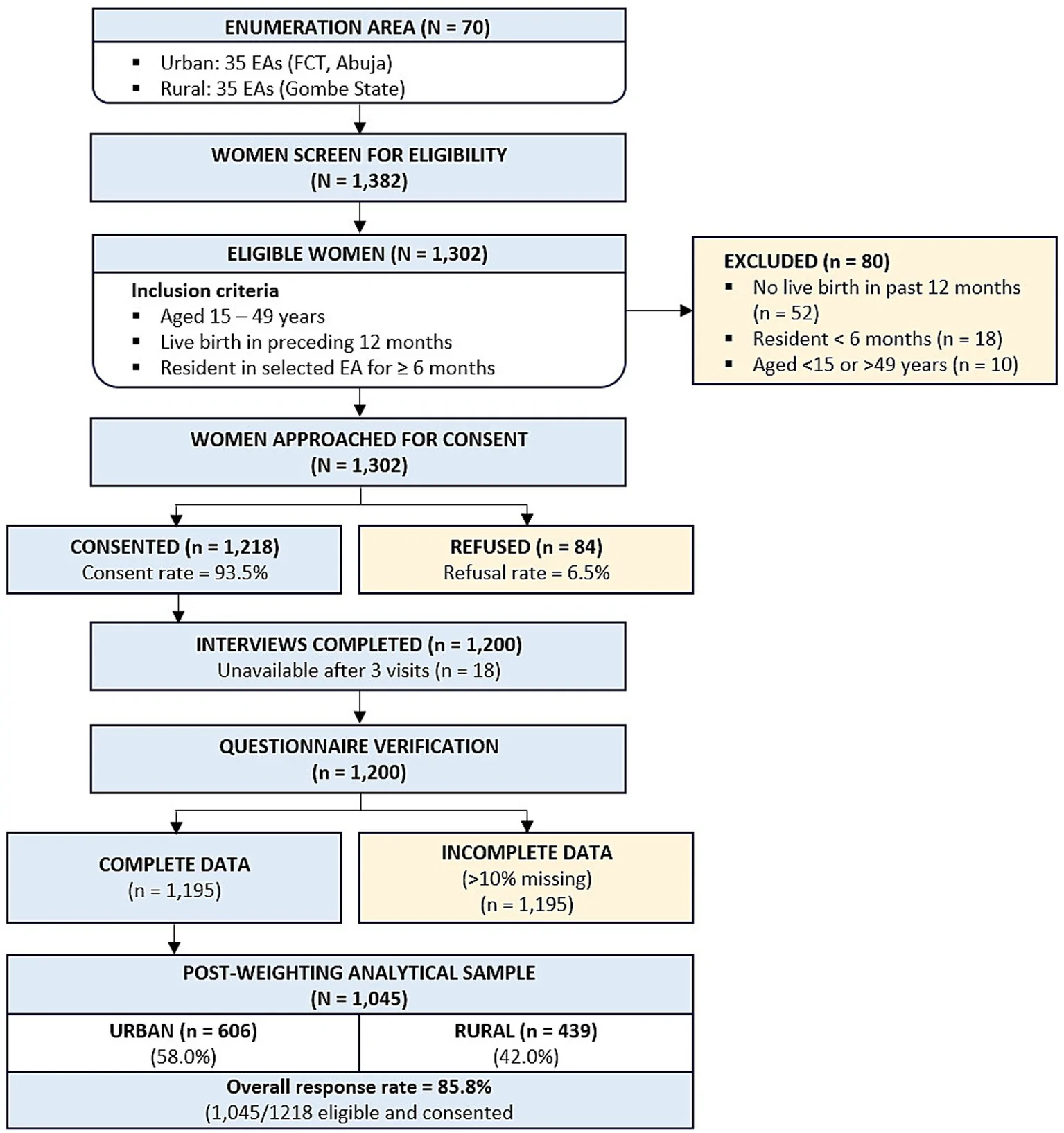
Participant flow diagram.

A total of 1,382 women were identified as potentially eligible across the 70 enumeration areas (35 urban in FCT, 35 rural in Gombe). Of these, 1,302 met the eligibility criteria and were approached for participation. After excluding 84 women who refused consent (6.5%) and 18 who were unavailable after three visits (1.4%), the achieved sample was 1,200. An additional 5 questionnaires were excluded due to incomplete data (>10% missing), yielding a final analytical sample of 1,195. However, after applying sampling weights and verifying eligibility for the 12-month recall period, the weighted analytical sample was 1,045 (606 urban, 439 rural). The overall response rate among eligible women was 85.8% (1,045/1,218). A participant flow diagram is provided in Figure 2.

### 3.2 Demographic characteristics of respondents

While urban and rural respondents were comparable in age, marital status, religion, and household size (*p* > 0.05), significant differences favoured urban women in education, employment, income, and parity (Table 2).

**Table 2:** Sociodemographic characteristics of respondents by place of residence (*N* = 1,045).

Variable	Category	Urban *n* (%) (*n* = 606)	Rural *n* (%) (*n* = 439)	*χ* ^2^	*p*-value
Age (years)	<20 years	65 (10.7)	47 (10.7)	3.92	0.271
	20–29 years	262 (43.2)	208 (47.4)		
	30–39 years	207 (34.2)	147 (33.5)		
	≥40 years	72 (11.9)	37 (8.4)		
Marital status	Single	53 (8.7)	35 (8.0)	0.54	0.762
	Married	494 (81.5)	356 (81.1)		
	Separated/Divorced/Widowed	59 (9.7)	48 (10.9)		
Religion	Christianity	280 (46.2)	210 (47.8)	3.81	0.283
	Islam	299 (49.3)	205 (46.7)		
	Traditional religion	27 (4.5)	24 (5.5)		
Highest level of education	No formal education	32 (5.3)	82 (18.7)	48.27	<0.001*
	Primary	78 (12.9)	110 (25.1)		
	Secondary	248 (40.9)	187 (42.6)		
	Tertiary	248 (40.9)	60 (13.7)		
Employment status	Employed	189 (31.2)	50 (11.4)	56.84	<0.001*
	Self-employed	243 (40.1)	137 (31.2)		
	Unemployed	174 (28.7)	252 (57.4)		
Number of household members	1–4 members	145 (23.9)	110 (25.1)	0.21	0.902
	5–8 members	343 (56.6)	243 (55.4)		
	≥9 members	118 (19.5)	86 (19.6)		
Monthly household income	< ₦50,000	174 (28.7)	279 (63.6)	92.61	<0.001*
	₦50,000–₦100,000	252 (41.6)	116 (26.4)		
	> ₦100,000	180 (29.7)	44 (10.0)		
Number of children	1 child	146 (24.1)	109 (24.8)	11.35	0.003*
	2–4 children	392 (64.7)	250 (56.9)		
	≥5 children	68 (11.2)	80 (18.2)		

*Statistically significant at *p* < 0.05.

### 3.3 Urban–rural comparison of maternal healthcare knowledge

Urban women demonstrated significantly higher levels of maternal healthcare knowledge than rural women across all 15 individual knowledge items (all *p* < 0.001; detailed item-wise comparison in Supplementary Table S1). Consequently, as shown in Table 3, the proportion of women with good overall knowledge was substantially higher in urban areas, while poor knowledge was nearly five times more common among rural women (*p* < 0.001).

**Table 3:** Comparison of overall knowledge scores by residence.

Knowledge category	Urban *n* (%) (*n* = 606)	Rural *n* (%) (*n* = 439)	*χ* ^2^	*p*-value
Poor knowledge (0–5)	28 (4.6)	96 (21.9)	92.47	<0.001*
Moderate knowledge (6–10)	176 (29.0)	231 (52.6)		
Good knowledge (11–15)	402 (66.3)	112 (25.5)		

*Statistically significant at *p* < 0.05.

### 3.4 Urban–rural comparison of maternal healthcare attitudes

Urban women held more positive attitudes toward maternal healthcare than rural women across all 11 Likert-scale statements (detailed full Likert comparison in Supplementary Table S2; binary attitude comparison in Supplementary Table S3). As shown in Table 4, the proportion of women with a positive overall attitude was substantially higher among urban respondents, while negative attitude was nearly four times more common in rural areas (*p* < 0.001).

**Table 4:** Comparison of overall attitude scores by residence (N = 1,045).

Attitude category	Urban *n* (%) (*n* = 606)	Rural *n* (%) (*n* = 439)	*χ* ^2^	*p*-value
Negative attitude	78 (12.9)	211 (48.1)	156.42	<0.001*
Positive attitude	528 (87.1)	228 (51.9)		

*Statistically significant at *p* < 0.05.

### 3.5 Urban–rural comparison of maternal healthcare practices

Urban women demonstrated significantly better uptake of all recommended maternal health practices than rural women (detailed practice comparisons in Supplementary Table S4). As shown in Table 5, the overall practice score was substantially higher among urban women, with the majority of urban respondents achieving good practice compared to less than one-quarter of rural respondents. Poor practice was more than four times higher in rural areas (*p* < 0.001).

**Table 5:** Comparison of overall maternal healthcare practice scores by residence (*N* = 1,045).

Practice category	Urban *n* (%) (*n* = 606)	Rural *n* (%) (*n* = 439)	*χ* ^2^	*p*-value
Poor practice (0–5)	42 (6.9)	128 (29.2)	118.76	<0.001*
Fair practice (6–9)	214 (35.3)	206 (46.9)		
Good practice (10–14)	350 (57.8)	105 (23.9)		

*Statistically significant at *p* < 0.05.

### 3.6 Factors associated with good maternal healthcare knowledge

Hierarchical logistic regression was used to examine the independent contribution of each SEM level to good maternal healthcare knowledge. Block 1 (individual-level: age, education, employment, income) explained 18.4% of the variance (Nagelkerke *R*^2^ = 0.184). Adding Block 2 (interpersonal-level: marital status) did not significantly improve the model (Δ*R*^2^ = 0.003, *p* = 0.312). Block 3 (institutional-level: affordability, respect, safety) significantly improved the model (Δ*R*^2^ = 0.087, *p* < 0.001), and Block 4 (community-level: place of residence) further significantly improved the model (Δ*R*^2^ = 0.062, *p* < 0.001). The final model explained 33.6% of the variance (Nagelkerke *R*^2^ = 0.336).

In bivariate analysis (Supplementary Table S5), urban residence, age ≥30 years, secondary/tertiary education, employment, higher income, ANC attendance, perceived affordability, respectful healthcare workers, and feeling safe at health facilities were all significantly associated with good knowledge (all *p* < 0.05). Multivariable logistic regression (Table 6) identified six independent predictors of good knowledge. The model showed acceptable discrimination (AUC = 0.78, 95% CI: 0.75–0.81).

**Table 6:** Hierarchical logistic regression analysis of predictors of good maternal healthcare knowledge among respondents (*N* = 1,045).

Model summary				
Block	Variables included	Nagelkerke *R*^2^	Δ*R*^2^	*p*-value for Δ*R*^2^
Block 1	Age, education, employment, income	0.184	–	–
Block 2	+ Marital status	0.187	0.003	0.312
Block 3	+ Affordability, respect, safety	0.274	0.087	<0.001*
Block 4	+ Place of residence	0.336	0.062	<0.001*

Final model (Block 4)				
Variable	Category	AOR	95% CI	*p*-value
Place of residence	Urban vs. Rural	3.84	2.67–5.53	<0.001*
Age group	≥30 years vs. < 30 years	1.36	0.98–1.89	0.067
Highest level of education	Secondary/Tertiary vs. None/Primary	3.16	2.19–4.55	<0.001*
Employment status	Employed/Self-employed vs. Unemployed	1.41	0.99–2.01	0.056
Monthly household income	≥ ₦50,000 vs. < ₦50,000	1.72	1.21–2.45	0.003*
Are maternal healthcare services affordable?	Yes vs. No	1.68	1.16–2.43	0.006*
Are healthcare workers respectful during ANC/PNC visits?	Yes vs. No	1.39	0.95–2.04	0.089
Do you feel safe attending health facilities?	Yes vs. No	1.52	1.01–2.29	0.044*

*Statistically significant at *p* < 0.05. AUC = 0.78 (95% CI: 0.75–0.81).

### 3.7 Factors associated with positive maternal healthcare attitude

Hierarchical logistic regression for positive attitude showed that Block 1 (individual-level) explained 12.1% of the variance (Nagelkerke *R*^2^ = 0.121). Adding Block 2 (interpersonal-level) did not significantly improve the model (Δ*R*^2^ = 0.004, *p* = 0.287). Block 3 (institutional-level) significantly improved the model (Δ*R*^2^ = 0.113, *p* < 0.001), and Block 4 (community-level) further significantly improved the model (Δ*R*^2^ = 0.071, *p* < 0.001). Adding good knowledge to the final model significantly improved the model (Δ*R*^2^ = 0.027, *p* < 0.001). The final model explained 33.6% of the variance (Nagelkerke *R*^2^ = 0.336).

In bivariate analysis (Supplementary Table S6), urban residence, age ≥30 years, married status, higher education, employment, higher income, good knowledge, perceived affordability, respectful healthcare workers, and feeling safe at health facilities were significantly associated with positive attitude (all *p* < 0.05). Multivariable logistic regression (Table 7) identified six independent predictors of positive attitude. The model showed good discrimination (AUC = 0.82, 95% CI: 0.79–0.85).

**Table 7:** Hierarchical logistic regression analysis of predictors of positive maternal healthcare attitude among respondents (*N* = 1,045).

Model summary				
Block	Variables included	Nagelkerke *R*^2^	Δ*R*^2^	*p*-value for Δ*R*^2^
Block 1	Age, education, employment, income	0.121	-	-
Block 2	+ Marital status	0.125	0.004	0.287
Block 3	+ Affordability, respect, safety	0.238	0.113	<0.001*
Block 4	+ Place of residence	0.309	0.071	<0.001*
Final model	+ Good knowledge	0.336	0.027	<0.001*

Final model				
Variable	Category	AOR	95% CI	*p*-value
Place of residence	Urban vs. Rural	3.41	2.38–4.89	<0.001*
Age group	≥30 years vs. < 30 years	1.28	0.92–1.78	0.141
Marital status	Married vs. Others	1.37	0.92–2.04	0.118
Highest level of education	Secondary/Tertiary vs. None/Primary	2.11	1.46–3.05	<0.001*
Employment status	Employed/Self-employed vs. Unemployed	1.29	0.89–1.87	0.173
Monthly household income	≥ ₦50,000 vs. < ₦50,000	1.54	1.05–2.27	0.028*
Good maternal healthcare knowledge	Yes vs. No	4.28	3.03–6.05	<0.001*
Are maternal healthcare services affordable?	Yes vs. No	1.43	0.98–2.08	0.061
Are healthcare workers respectful during ANC/PNC visits?	Yes vs. No	1.97	1.35–2.89	<0.001*
Do you feel safe attending health facilities?	Yes vs. No	1.72	1.18–2.51	0.005*

*Statistically significant at *p* < 0.05. AUC = 0.82 (95% CI: 0.79–0.85).

### 3.8 Factors associated with good maternal healthcare practice

Hierarchical logistic regression for good practice showed that Block 1 (individual-level) explained 11.8% of the variance (Nagelkerke *R*^2^ = 0.118). Adding Block 2 (interpersonal-level) did not significantly improve the model (Δ*R*^2^ = 0.003, *p* = 0.345). Block 3 (institutional-level) significantly improved the model (Δ*R*^2^ = 0.098, *p* < 0.001), and Block 4 (community-level) further significantly improved the model (Δ*R*^2^ = 0.056, *p* < 0.001). Adding good knowledge and positive attitude to the final model significantly improved the model (Δ*R*^2^ = 0.068, *p* < 0.001). The final model explained 34.3% of the variance (Nagelkerke *R*^2^ = 0.343).

In bivariate analysis (Supplementary Table S7), urban residence, age ≥30 years, married status, higher education, employment, higher income, good knowledge, positive attitude, perceived affordability, respectful healthcare workers, and feeling safe at health facilities were significantly associated with good practice (all *p* < 0.05). Multivariable logistic regression (Table 8) identified seven independent predictors of good practice. The model showed acceptable discrimination (AUC = 0.79, 95% CI: 0.76–0.82).

**Table 8:** Hierarchical logistic regression analysis of predictors of good maternal healthcare practice among respondents (*N* = 1,045).

Model summary				
Block	Variables Included	Nagelkerke *R*^2^	Δ*R*^2^	*p*-value for Δ*R*^2^
Block 1	Age, education, employment, income	0.118	–	–
Block 2	+ Marital status	0.121	0.003	0.345
Block 3	+ Affordability, respect, safety	0.219	0.098	<0.001*
Block 4	+ Place of residence	0.275	0.056	<0.001*
Final model	+ Good knowledge, positive attitude	0.343	0.068	<0.001*

Final model				
Variable	Category	AOR	95% CI	*p*-value
Place of residence	Urban vs. Rural	2.84	1.97–4.09	<0.001*
Age group	≥30 years vs. < 30 years	1.31	0.94–1.83	0.111
Marital status	Married vs. Others	1.29	0.87–1.92	0.204
Highest level of education	Secondary/Tertiary vs. None/Primary	1.88	1.29–2.74	0.001*
Employment status	Employed/Self-employed vs. Unemployed	1.42	0.98–2.05	0.062
Monthly household income	≥ ₦50,000 vs. < ₦50,000	1.57	1.08–2.29	0.019*
Good maternal healthcare knowledge	Yes vs. No	3.91	2.74–5.57	<0.001*
Positive maternal healthcare attitude	Yes vs. No	3.27	2.27–4.70	<0.001*
Are maternal healthcare services affordable?	Yes vs. No	1.81	1.24–2.64	0.002*
Are healthcare workers respectful during ANC/PNC visits?	Yes vs. No	1.69	1.15–2.48	0.008*
Do you feel safe attending health facilities?	Yes vs. No	1.41	0.95–2.10	0.087

*Statistically significant at *p* < 0.05. AUC = 0.79 (95% CI: 0.76–0.82).

## 4 Discussion

This comparative cross-sectional study examined urban–rural disparities in maternal healthcare KAP among postpartum women in Abuja and Gombe States, Nigeria, using the SEM as a guiding theoretical framework. The central finding is a systematic and substantial urban advantage across all SEM levels. Urban women demonstrated considerably higher levels of knowledge, more positive attitudes, and better uptake of recommended maternal practices than their rural counterparts. Urban residence, conceptualised as a community-level factor within SEM, was strongly associated with good knowledge (AOR = 3.84), positive attitudes (AOR = 3.41), and good practice (AOR = 2.84). Furthermore, consistent with the KAP model, a hierarchical knowledge-attitude-practice pathway was evident, where knowledge and attitudes were associated with practices, though the cross-sectional design precludes causal inference.

The hierarchical regression analysis revealed that individual-level factors explained the largest proportion of variance for all three outcomes (knowledge: 18.4%, attitude: 12.1%, practice: 11.8%), followed by institutional-level factors (knowledge: 8.7%, attitude: 11.3%, practice: 9.8%), and community-level factors (knowledge: 6.2%, attitude: 7.1%, practice: 5.6%). Interpersonal-level factors did not significantly improve any of the models. These findings suggest that while individual characteristics are foundational, institutional and community-level factors contribute substantially to maternal KAP, supporting the multi-level approach of the SEM framework.

### 4.1 Urban–rural disparities in maternal healthcare knowledge

The observation that urban women consistently outperformed rural women across all 15 knowledge domains is an important public health signal. It indicates that the information deficit in rural areas is not limited to isolated topics but represents a systematic gap in access to maternal health education. A nationwide analysis by Adewuyi et al. directly examined antenatal care knowledge and reported that rural residents in northern Nigeria had significantly lower receipt of ANC components compared to urban residents, with the largest gaps observed for knowledge of danger signs (urban: 62% vs. rural: 38%) and recommended supplementation (urban: 55% vs. rural: 29%) (25). Our study extends this evidence by showing that the knowledge gap is universal across specific items including danger signs (urban: 71% vs. rural: 31%), supplementation (urban: 68% vs. rural: 28%), and IPTp (urban: 59% vs. rural: 22%), suggesting that the deficit is foundational rather than situational.

At the individual level within the SEM framework, the knowledge gap likely reflects lower mass media exposure, lower female literacy, and fewer community health workers. At the institutional level, under-resourced rural facilities cannot provide consistent, high-quality health education. At the community level, rural social norms may not prioritise formal maternal health information seeking. A systematic review of maternal health literacy in low-income countries found that rural women had consistently lower health literacy scores than urban women, mediated largely by educational attainment and access to information sources (6). The implication is that rural health education programmes must be delivered through accessible, low-literacy channels such as radio messaging, pictorial aids, and community health worker home visits, rather than relying on written materials or passive information transfer at facilities.

It is important to note that while employment status (AOR = 1.41, *p* = 0.056) and healthcare worker respect (AOR = 1.39, *p* = 0.089) showed positive associations with good knowledge, these findings did not reach statistical significance. We therefore interpret these as suggestive trends requiring further investigation in larger samples, rather than as definitive predictors of knowledge.

### 4.2 Urban–rural disparities in maternal healthcare attitudes

Urban women held more positive attitudes toward facility delivery, vaccination, supplementation, and rejecting traditional healers. Good knowledge was strongly associated with positive attitudes (AOR = 4.28, *p* < 0.001), consistent with the theoretical KAP model. However, because our data are cross-sectional, we cannot determine whether knowledge precedes attitude, attitude precedes knowledge, or both are influenced by a third factor such as education or prior healthcare experiences. At the institutional level, rural women’s less favourable attitudes may also reflect negative prior experiences with formal health facilities, including disrespectful care, long waiting times, and stock-outs of essential supplies, all of which can erode trust even when knowledge is present. This aligns with the SEM framework’s emphasis on institutional factors shaping health behaviours.

While age (AOR = 1.28, *p* = 0.141), marital status (AOR = 1.37, *p* = 0.118), employment status (AOR = 1.29, *p* = 0.173), and perceived affordability (AOR = 1.43, *p* = 0.061) showed positive associations with positive attitudes, these findings were not statistically significant. In contrast, the strong independent effects of education (AOR = 2.11, *p* < 0.001), income (AOR = 1.54, *p* = 0.028), knowledge (AOR = 4.28, *p* < 0.001), healthcare worker respect (AOR = 1.97, *p* < 0.001), and perceived safety (AOR = 1.72, *p* = 0.005) were statistically significant and represent robust predictors of positive attitudes.

A cluster randomised trial in rural Ethiopia by Gebretsadik and colleagues found that improving knowledge through social and behaviour change communication significantly improved attitudes and institutional childbirth rates, providing experimental evidence for the knowledge-to-attitude direction (26). In contrast, a Nigerian study on breastfeeding by Balogun and colleagues found no urban–rural attitude difference (27). However, that study is now 8 years old and examined a different domain (breastfeeding) that is a universal, home-based practice requiring minimal facility interaction. Attitudes toward facility-based care are inherently more context dependent and more likely to be shaped by access and quality of services. The comparison is therefore imperfect, and the contrasting findings likely reflect domain-specific effects rather than methodological differences. Future research should examine whether the urban–rural attitude gap varies systematically across different maternal health behaviours.

### 4.3 Urban–rural disparities in maternal healthcare practices

The practice gap was the most consequential finding. Urban women had substantially higher uptake of recommended maternal practices, including early ANC initiation, adequate visit frequency, supplementation, IPTp, tetanus toxoid vaccination, and facility-based delivery. Rural women experienced danger signs more frequently, indicating that inadequate practice translates directly into morbidity. Good knowledge (AOR = 3.91, *p* < 0.001) and positive attitude (AOR = 3.27, p < 0.001) were strong correlates of good practice. Yet the association between knowledge and practice was somewhat attenuated compared to the knowledge-attitude association, suggesting that other factors such as cost, distance, and fear of mistreatment may block the final step from intention to behaviour.

Applying the SEM, the observed practice gap reflects barriers operating across multiple ecological levels, including individual-level factors such as lower education and household income, which limit health literacy and financial resources; interpersonal-level factors, where limited household decision-making autonomy may constrain healthcare-seeking; institutional-level factors, including poor affordability of services, disrespectful care, and safety concerns that discourage facility utilization; and community-level factors, where rural residence further exacerbates these challenges through geographic isolation and weaker health infrastructure.

While age, marital status, employment status, and perceived safety showed positive associations with good practice, these findings were not statistically significant. The significant predictors of good practice were place of residence (AOR = 2.84, *p* < 0.001), education (AOR = 1.88, *p* = 0.001), income (AOR = 1.57, *p* = 0.019), knowledge (AOR = 3.91, *p* < 0.001), attitude (AOR = 3.27, *p* < 0.001), affordability (AOR = 1.81, *p* = 0.002), and healthcare worker respect (AOR = 1.69, *p* = 0.008).

This pattern is consistent with a Lagos study by Olubodun and colleagues, where despite near universal antenatal care attendance, many low-income women still delivered outside facilities due to cost and disrespectful care (28). A qualitative study in the same Gombe State by Alhaji and colleagues identified direct and indirect costs (specifically transport costs and user fees averaging ₦5,000–₦10,000 per visit), poor service quality (including stock-outs of essential medicines in 43% of facilities), and traditional norms (preference for home delivery in 38% of respondents) as persistent barriers (29). Our findings add that these barriers are compounded by lower knowledge and less favourable attitudes, creating a triple disadvantage. The implication is that interventions must simultaneously address knowledge through education, attitudes through respectful care, and access through subsidies or insurance. A single-domain intervention is unlikely to close the urban–rural practice gap.

### 4.4 Predictors of good maternal healthcare KAP

Women with secondary or tertiary education had substantially higher odds of good knowledge (AOR = 3.16), positive attitudes (AOR = 2.11), and good practice (AOR = 1.88). Higher household income similarly predicted all three outcomes (AORs: 1.72, 1.54, and 1.57 respectively). Education likely enhances health literacy, self-efficacy, and the ability to navigate the health system. Income removes practical obstacles such as transport costs, user fees, and the opportunity cost of time. These findings align with a cross-national analysis by Bobo and colleagues across 25 African countries, which found that education, mass media exposure, and wealth are consistent predictors of completing the continuum of care (30).

Within the SEM framework, education and income are individual-level factors that interact with institutional and community-level factors to shape maternal KAP. The strong association between education and maternal KAP in our study raises the possibility that unmeasured confounding by husband’s education or household decision-making power may explain some of the observed effects. We did not collect data on these variables, which is a limitation. Nevertheless, the magnitude and consistency of the education effect across all three outcomes suggest that investments in girls’ secondary education and poverty reduction are not long-term aspirations but essential, concurrent components of any strategy to reduce maternal health inequities in rural Nigeria.

### 4.5 Health system factors and their influence on maternal KAP

Perceived affordability, respectfulness of healthcare workers, and feeling safe at health facilities had distinct patterns of association. Affordability was associated with knowledge (AOR = 1.68) and practice (AOR = 1.81) but not attitude, whereas respectfulness was associated with attitude (AOR = 1.97) and practice (AOR = 1.69), and safety was associated with knowledge (AOR = 1.52) and attitude (AOR = 1.72). This pattern suggests that financial constraints affect the practical ability to act without necessarily changing how women feel about care. Respectful care, however, directly influences emotional orientation, which then shapes both attitude and return behaviour.

At the institutional level within the SEM framework, these findings highlight that health system quality is not merely a practical concern but a determinant of attitudes and subsequent practices. A study in South–South Nigeria by Ekpenyong found that negative staff attitudes and breaches of confidentiality were major barriers, leading women to turn to traditional birth attendants who offered compassion even at the cost of safety (31). That finding mirrors our result that respect independently predicts attitude. The implication is that policy must treat affordability and quality of care as complementary investments. Removing user fees without improving respectful care will not close the attitude gap, and improving staff training without addressing cost will not close the practice gap.

### 4.6 Application of the social ecological model

Applying the SEM illuminates how factors across multiple levels interact to produce urban–rural disparities. At the individual level, education and income emerged as the strongest predictors, suggesting that health literacy and financial resources are foundational to maternal KAP. At the institutional level, perceived affordability, respectfulness of healthcare workers, and safety concerns influenced attitudes and practices, reflecting the quality of the health system in shaping care-seeking behaviour. At the community level, urban residence captured a constellation of advantages, better infrastructure, greater information access, and stronger social norms favouring facility-based care, that compounded individual-level benefits.

The hierarchical regression analysis provided empirical support for the SEM framework, demonstrating that each ecological level contributed independently to maternal KAP. Individual-level factors consistently explained the largest proportion of variance (11.8–18.4%), followed by institutional-level factors (8.7–11.3%) and community-level factors (5.6–7.1%). Interpersonal-level factors did not significantly improve any of the models, suggesting that in this context, individual, institutional, and community factors may be more influential than marital status or household-level dynamics. The finding that institutional factors explained substantial variance after controlling for individual characteristics underscores the importance of health system quality in shaping maternal KAP.

The KAP model’s hypothesised hierarchical pathway was also supported: good knowledge was the strongest predictor of positive attitude (AOR = 4.28), and both knowledge (AOR = 3.91) and attitude (AOR = 3.27) independently predicted good practice. This suggests that interventions targeting knowledge may have downstream effects on attitudes and practices, but that direct interventions on attitudes and institutional factors are also necessary.

The consistent urban advantage across all SEM levels suggests that interventions must be multi-level: policies promoting girls’ secondary education (individual), health system reforms ensuring respectful care and affordability (institutional), and community-based health education addressing norms and information access (community). Single-level interventions are unlikely to close the urban–rural gap, as disparities are reinforced across multiple ecological levels.

### 4.7 Strengths and limitations

#### 4.7.1 Strengths

This study has several methodological strengths. The large community-based sample of over 1000 women, with sampling weights applied to account for the multi-stage cluster design, enhances generalisability, while the design effect for the primary outcome (good practice) was 1.52 and the effective sample size approximately 687, indicating adequate precision. The use of validated KAP instruments with robust content validity (S-CVI/UA = 0.89, S-CVI/Ave = 0.92) and good to excellent internal consistency (Cronbach’s *α* = 0.84–0.88) supports measurement reliability, and the comparative design directly contrasting two geographically distinct settings using NPC-classified urban and rural enumeration areas provided a robust framework for understanding urban–rural disparities while avoiding ecological fallacy. The *a priori* specification of variable selection based on the Social Ecological Model, combined with acceptable model fit statistics (AUC = 0.78–0.82), strengthens analytical rigour, and the integration of the SEM-KAP theoretical framework provides a comprehensive lens for interpreting findings and guiding recommendations. The hierarchical regression approach, with variables entered in blocks according to SEM levels, allowed us to quantify the independent contribution of each ecological level and demonstrated that institutional and community factors explain substantial variance beyond individual characteristics. Furthermore, the explicit scoring of ‘Do not Know’ responses as incorrect (0) for knowledge items represents a methodological strength, as it conservatively estimates knowledge and avoids overestimation that could occur if such responses were excluded or treated as correct, ensuring that only women with demonstrable knowledge are classified as having good knowledge. Finally, the ethical practice of obtaining assent from minors aged 15–17 years alongside parental/guardian consent is another strength, particularly in the culturally sensitive context of northern Nigeria, as this dual consent process respects the autonomy of adolescent participants while acknowledging the authority of parents and guardians, and aligns with ethical guidelines for research involving minors in sensitive health topics.

#### 4.7.2 Limitations

Several limitations must be acknowledged. First, non-response was higher in rural areas (achieved 439 vs. target 600) due to seasonal migration and security constraints in parts of Gombe State; if non-respondents were systematically poorer or had lower KAP than respondents, our estimates would be conservative, underestimating the true rural disadvantage, and while we attempted to mitigate this through sampling weights, residual non-response bias may remain. Second, social desirability bias may have influenced responses, particularly for attitude statements and self-reported practices, though we minimised this through private interviewing locations, trained female enumerators, and confidentiality assurances; nevertheless, some over-reporting of positive attitudes and practices may persist, potentially biasing our estimates toward the null, although the consistency of our findings with objective health service data from the NDHS (3) suggests that social desirability bias, if present, did not substantially distort the overall patterns. Third, the 12-month recall period is subject to recall bias, consistent with Demographic and Health Survey methodology, and while we recommend that future studies stratify analyses by time since delivery (<6 months vs. 6–12 months) to assess recall accuracy, we were unable to conduct this stratification due to sample size limitations in the rural stratum.

Fourth, despite adjusting for key sociodemographic and health system variables, residual confounding by unmeasured variables remains possible; we did not collect data on husband’s education, household decision-making power, previous pregnancy outcomes, distance to the nearest facility, or facility readiness, all of which could explain some of the observed associations, and future studies should include these variables to more fully characterise the pathways linking urban residence to maternal KAP. Fifth, the rural sample was limited to Gombe State, which may not represent all rural areas in Nigeria, particularly those in the South–South or South-East regions, while Abuja as an urban centre may have superior health infrastructure compared to other urban areas, potentially overestimating the urban advantage; furthermore, the comparison of urban EAs in FCT with rural EAs in Gombe conflates urban–rural differences with state-level differences, including variations in health policies, funding allocation, and socio-cultural characteristics, and we therefore interpret our findings as reflecting disparities between an urban setting (FCT) and a rural setting (Gombe) rather than a pure urban–rural comparison within the same administrative context, a distinction that should be considered when generalising our findings to other Nigerian settings, and future multi-state studies with urban and rural samples within each state are needed to disentangle urban–rural differences from state-level effects.

Sixth, we relied on the 2018 NPC census frame for urban–rural classification, and given rapid urbanisation in Nigeria, particularly in peri-urban areas around the FCT, it is possible that some areas classified as rural in 2018 have experienced significant urban growth in the intervening years, potentially resulting in some misclassification, though such misclassification would likely attenuate rather than exaggerate the observed urban–rural differences, and future studies should consider using more recent census data or alternative classification approaches. Seventh, the cross-sectional design precludes causal inference, as temporality between knowledge, attitudes, and practices cannot be definitively established, and we have therefore used cautious language throughout, describing associations rather than causal effects. Finally, the attitude dichotomisation using an 80% cut-off, while *a priori*, may not be directly comparable to studies using different cut-offs, and while we did not conduct sensitivity analyses with alternative cut-offs (e.g., 70% or median split), we acknowledge this as a limitation and recommend such analyses in future research. These limitations do not invalidate the core conclusions but highlight areas for future research, including longitudinal studies to establish temporal sequences, mixed-methods designs to explore how attitudes are formed and changed in rural settings, and inclusion of supply-side and household-level variables to more fully characterise the pathways linking urban residence to maternal KAP.

## 5 Conclusion

This study demonstrates that substantial urban–rural disparities in maternal healthcare knowledge, attitudes, and practices persist in Nigeria, driven by factors across multiple levels of the Social Ecological Model: individual-level factors (education, income), institutional-level factors (affordability, disrespectful care, safety concerns), and community-level factors (urban residence). The hierarchical regression analysis provided empirical support for the SEM framework, demonstrating that individual-level factors explained the largest proportion of variance (11.8–18.4%), followed by institutional-level factors (8.7–11.3%) and community-level factors (5.6–7.1%). The findings are consistent with the KAP model’s hypothesised hierarchical pathway, where knowledge was strongly associated with attitudes, and both knowledge and attitudes were independently associated with practices, providing theoretical support for this integrated SEM-KAP framework in guiding multi-level interventions. Based on our findings and theoretical grounding, interventions that promote secondary education for girls (individual level), deliver community-based health education using accessible media such as radio and mobile phones (community level), eliminate financial barriers through health insurance or targeted subsidies (institutional level), train healthcare workers in respectful maternity care (institutional level), and improve facility safety (institutional level) are likely to reduce disparities in rural Nigeria; however, without concurrent action on socioeconomic determinants and health system quality across all ecological levels, the urban–rural gap will likely remain. Future research should include longitudinal studies to establish temporal sequences and test causal pathways within the SEM-KAP framework, implementation science to design, deliver, and evaluate multi-component interventions aligned with the SEM framework, mixed-methods designs to explore how attitudes are formed and changed in rural settings including the role of social norms and healthcare experiences, and inclusion of supply-side variables (facility readiness, provider density, distance to care) and household-level variables (husband’s education, decision-making autonomy, previous pregnancy outcomes) to more fully characterise the pathways linking urban residence to maternal KAP.

## Data Availability

The original contributions presented in the study are included in the article/Supplementary material, further inquiries can be directed to the corresponding author.
