# Supplementary material for: Urban–rural disparities in maternal healthcare knowledge, attitudes, and practices among women with a recent live birth in Abuja and Gombe states, Nigeria
Source: Front Public Health. 2026 Aug 26;14:1909190. doi: 10.3389/fpubh.2026.1909190 (PMC13553966; doi:10.3389/fpubh.2026.1909190)
Supplement: Supplementary file 1 [file Table_1.DOCX]

**Supplementary Results**

**Table S1. Comparison of Maternal Healthcare Knowledge Between Urban and Rural Respondents**

| **Knowledge Variable** | **Category** | **Urban n (%)**  **(n = 606)** | **Rural n (%)**  **(n = 439)** | **χ²** | **p-value** |
| --- | --- | --- | --- | --- | --- |
| Have you heard about antenatal care (ANC)? | Yes | 592 (97.7) | 402 (91.6) | 19.84 | <0.001* |
|  | No | 14 (2.3) | 37 (8.4) |  |  |
| How many ANC visits are recommended during pregnancy? | <4 visits | 61 (10.1) | 107 (24.4) | 33.26 | <0.001* |
|  | 4–7 visits | 320 (52.8) | 203 (46.2) |  |  |
|  | ≥8 visits | 182 (30.0) | 65 (14.8) |  |  |
|  | I don’t know | 43 (7.1) | 64 (14.6) |  |  |
| Where should ANC services ideally be obtained? | Health facility | 575 (94.9) | 372 (84.7) | 30.54 | <0.001* |
|  | Traditional birth home | 31 (5.1) | 67 (15.3) |  |  |
| Do you know any danger signs during pregnancy? | Yes | 548 (90.4) | 339 (77.2) | 34.19 | <0.001* |
|  | No | 58 (9.6) | 100 (22.8) |  |  |
| Which of the following are danger signs during pregnancy? # | Correct identification | 503 (91.8) | 286 (84.4) | 11.72 | 0.001* |
|  | Incorrect identification | 45 (8.2) | 53 (15.6) |  |  |
| Are laboratory investigations important during pregnancy? | Yes | 584 (96.4) | 385 (87.7) | 28.27 | <0.001* |
|  | No | 22 (3.6) | 54 (12.3) |  |  |
| Which laboratory tests are commonly done during pregnancy? ## | Correct identification | 526 (90.1) | 297 (77.1) | 29.54 | <0.001* |
|  | Incorrect identification | 58 (9.9) | 88 (22.9) |  |  |
| Should tetanus toxoid vaccine be taken during pregnancy? | Yes | 585 (96.5) | 376 (85.6) | 43.75 | <0.001* |
|  | No | 21 (3.5) | 63 (14.4) |  |  |
| How many doses of tetanus toxoid are recommended? | 1 dose | 72 (11.9) | 85 (19.4) | 39.10 | <0.001* |
|  | 2 doses | 471 (77.7) | 261 (59.5) |  |  |
|  | I don’t know | 63 (10.4) | 93 (21.2) |  |  |
| Have you heard about IPTp-SP for malaria prevention during pregnancy? | Yes | 533 (88.0) | 309 (70.4) | 49.47 | <0.001* |
|  | No | 73 (12.0) | 130 (29.6) |  |  |
| Have you heard about folic acid supplementation? | Yes | 576 (95.0) | 362 (82.5) | 43.06 | <0.001* |
|  | No | 30 (5.0) | 77 (17.5) |  |  |
| Have you heard about iron and calcium supplementation during pregnancy? | Yes | 561 (92.6) | 326 (74.3) | 63.51 | <0.001* |
|  | No | 45 (7.4) | 113 (25.7) |  |  |
| Is additional food intake necessary during pregnancy? | Yes | 582 (96.0) | 367 (83.6) | 48.57 | <0.001* |
|  | No | 24 (4.0) | 72 (16.4) |  |  |
| Is family planning important before pregnancy? | Yes | 507 (83.7) | 301 (68.6) | 32.96 | <0.001* |
|  | No | 99 (16.3) | 138 (31.4) |  |  |
| Can alcohol and smoking affect pregnancy outcomes? | Yes | 592 (97.7) | 401 (91.3) | 21.12 | <0.001* |
|  | No | 14 (2.3) | 38 (8.7) |  |  |

*Statistically significant at p < 0.05. # Percentages for “Which of the following are danger signs during pregnancy?” were calculated using only respondents who answered “Yes” to knowledge of danger signs during pregnancy (Urban n = 548; Rural n = 339). ## Percentages for “Which laboratory tests are commonly done during pregnancy?” were calculated using only respondents who answered “Yes” to the importance of laboratory investigations during pregnancy (Urban n = 584; Rural n = 385).

**Table S2. Comparison of Attitudes Towards Maternal Healthcare by Place of Residence Using Likert Scale Responses**

| **Attitude Statement** | **Residence** | **Strongly Agree**  **n (%)** | **Agree**  **n (%)** | **Neutral**  **n (%)** | **Disagree**  **n (%)** | **Strongly Disagree**  **n (%)** | **χ²** | **p-value** |
| --- | --- | --- | --- | --- | --- | --- | --- | --- |
| Regular ANC visits are important | Urban | 312 (51.5) | 239 (39.4) | 28 (4.6) | 19 (3.1) | 8 (1.3) | 56.34 | <0.001* |
|  | Rural | 154 (35.1) | 166 (37.8) | 62 (14.1) | 38 (8.7) | 19 (4.3) |  |  |
| Pregnant women should know danger signs during pregnancy | Urban | 325 (53.6) | 238 (39.3) | 21 (3.5) | 15 (2.5) | 7 (1.2) | 48.91 | <0.001* |
|  | Rural | 132 (30.1) | 209 (47.6) | 59 (13.4) | 49 (6.2) | 10 (2.3) |  |  |
| Laboratory investigations should be done during pregnancy | Urban | 336 (55.4) | 234 (38.6) | 18 (3.0) | 12 (2.0) | 6 (1.0) | 53.60 | <0.001* |
|  | Rural | 149 (33.9) | 198 (45.1) | 61 (13.9) | 42 (5.5) | 13 (3.0) |  |  |
| Tetanus vaccination is necessary during pregnancy | Urban | 330 (54.5) | 234 (38.6) | 20 (3.3) | 14 (2.3) | 8 (1.3) | 61.85 | <0.001* |
|  | Rural | 140 (31.9) | 193 (44.0) | 62 (14.1) | 47 (7.1) | 17 (3.9) |  |  |
| IPTp-SP helps prevent malaria during pregnancy | Urban | 316 (52.1) | 225 (37.1) | 33 (5.4) | 23 (3.8) | 9 (1.5) | 72.26 | <0.001* |
|  | Rural | 156 (35.5) | 142 (32.3) | 51 (11.6) | 36 (8.2) | 54 (12.3) |  |  |
| Iron and folic acid supplementation are important | Urban | 334 (55.1) | 234 (38.6) | 20 (3.3) | 12 (2.0) | 6 (1.0) | 63.47 | <0.001* |
|  | Rural | 136 (31.0) | 199 (45.3) | 59 (13.4) | 33 (7.5) | 12 (2.7) |  |  |
| Pregnant women should eat more nutritious food | Urban | 348 (57.4) | 229 (37.8) | 15 (2.5) | 9 (1.5) | 5 (0.8) | 47.64 | <0.001* |
|  | Rural | 159 (36.2) | 202 (46.0) | 41 (9.3) | 25 (5.7) | 12 (2.7) |  |  |
| Delivery should take place in a health facility | Urban | 351 (57.9) | 222 (36.6) | 14 (2.3) | 12 (2.0) | 7 (1.2) | 83.90 | <0.001* |
|  | Rural | 138 (31.4) | 191 (43.5) | 56 (12.8) | 36 (8.2) | 18 (4.1) |  |  |
| Traditional healers should not replace hospital care during pregnancy | Urban | 298 (49.2) | 228 (37.6) | 42 (6.9) | 27 (4.5) | 11 (1.8) | 109.73 | <0.001* |
|  | Rural | 97 (22.1) | 158 (36.0) | 72 (16.4) | 69 (15.7) | 43 (9.8) |  |  |
| Smoking and alcohol are harmful during pregnancy | Urban | 389 (64.2) | 200 (33.0) | 8 (1.3) | 5 (0.8) | 4 (0.7) | 28.81 | <0.001* |
|  | Rural | 188 (42.8) | 204 (46.5) | 18 (4.1) | 17 (3.9) | 12 (2.7) |  |  |
| Family planning counselling should be encouraged | Urban | 305 (50.3) | 231 (38.1) | 39 (6.4) | 21 (3.5) | 10 (1.7) | 84.48 | <0.001* |
|  | Rural | 101 (23.0) | 183 (41.7) | 71 (16.2) | 58 (13.2) | 26 (5.9) |  |  |

*Statistically significant at p < 0.05.

**Table S3. Comparison of Attitudes Towards Maternal Healthcare by Residence (N = 1,045)**

| **Attitude Statement** | **Category** | **Urban n (%) (n = 606)** | **Rural n (%) (n = 439)** | **χ²** | **p-value** |
| --- | --- | --- | --- | --- | --- |
| Regular ANC visits are important | Positive attitude | 551 (90.9) | 320 (72.9) | 56.34 | <0.001* |
|  | Negative attitude | 55 (9.1) | 119 (27.1) |  |  |
| Pregnant women should know danger signs during pregnancy | Positive attitude | 563 (92.9) | 341 (77.7) | 48.91 | <0.001* |
|  | Negative attitude | 43 (7.1) | 98 (22.3) |  |  |
| Laboratory investigations should be done during pregnancy | Positive attitude | 570 (94.1) | 347 (79.0) | 53.60 | <0.001* |
|  | Negative attitude | 36 (5.9) | 92 (21.0) |  |  |
| Tetanus vaccination is necessary during pregnancy | Positive attitude | 564 (93.1) | 333 (75.9) | 61.85 | <0.001* |
|  | Negative attitude | 42 (6.9) | 106 (24.1) |  |  |
| IPTp-SP helps prevent malaria during pregnancy | Positive attitude | 541 (89.3) | 298 (67.9) | 72.26 | <0.001* |
|  | Negative attitude | 65 (10.7) | 141 (32.1) |  |  |
| Iron and folic acid supplementation are important | Positive attitude | 568 (93.7) | 335 (76.3) | 63.47 | <0.001* |
|  | Negative attitude | 38 (6.3) | 104 (23.7) |  |  |
| Pregnant women should eat more nutritious food | Positive attitude | 577 (95.2) | 361 (82.2) | 47.64 | <0.001* |
|  | Negative attitude | 29 (4.8) | 78 (17.8) |  |  |
| Delivery should take place in a health facility | Positive attitude | 573 (94.6) | 329 (74.9) | 83.90 | <0.001* |
|  | Negative attitude | 33 (5.4) | 110 (25.1) |  |  |
| Traditional healers should not replace hospital care during pregnancy | Positive attitude | 526 (86.8) | 255 (58.1) | 109.73 | <0.001* |
|  | Negative attitude | 80 (13.2) | 184 (41.9) |  |  |
| Smoking and alcohol are harmful during pregnancy | Positive attitude | 589 (97.2) | 392 (89.3) | 28.81 | <0.001* |
|  | Negative attitude | 17 (2.8) | 47 (10.7) |  |  |
| Family planning counselling should be encouraged | Positive attitude | 536 (88.4) | 284 (64.7) | 84.48 | <0.001* |
|  | Negative attitude | 70 (11.6) | 155 (35.3) |  |  |

*Statistically significant at p < 0.05.

**Table S4. Comparison of Maternal Healthcare Practices Between Urban and Rural Respondents (N = 1,045)**

| **Practice Variable** | **Category** | **Urban n (%)** | **Rural n (%)** | **χ²** | **p-value** |
| --- | --- | --- | --- | --- | --- |
| Did you attend ANC during your last pregnancy? | Yes | 575 (94.9) | 363 (82.7) | 40.81 | <0.001* |
|  | No | 31 (5.1) | 76 (17.3) |  |  |
| At what stage of pregnancy did you first attend ANC? † | First trimester | 228 (39.7) | 77 (21.2) | 35.66 | <0.001* |
|  | Second trimester | 311 (54.1) | 262 (72.2) |  |  |
|  | Third trimester | 36 (6.3) | 24 (6.6) |  |  |
| How many ANC visits did you attend? † | <4 visits | 78 (13.6) | 133 (36.6) | 67.28 | <0.001* |
|  | 4–7 visits | 372 (64.7) | 170 (46.8) |  |  |
|  | ≥8 visits | 125 (21.7) | 60 (16.5) |  |  |
| Where did you receive ANC services? | Government hospital | 210 (34.7) | 157 (35.8) | 28.39 | <0.001* |
|  | PHC | 205 (33.8) | 213 (48.5) |  |  |
|  | Private hospital | 172 (28.4) | 37 (8.4) |  |  |
|  | Traditional birth home | 19 (3.1) | 32 (7.3) |  |  |
| Did you experience any danger signs during pregnancy? | Yes | 186 (30.7) | 232 (52.8) | 52.85 | <0.001* |
|  | No | 420 (69.3) | 207 (47.2) |  |  |
| Which danger signs did you experience? ‡ | Bleeding | 55 (29.6) | 83 (35.8) | 8.47 | 0.037* |
|  | Severe headache | 46 (24.7) | 72 (31.0) |  |  |
|  | Swollen legs | 38 (20.4) | 61 (26.3) |  |  |
|  | Fever | 39 (21.0) | 59 (25.4) |  |  |
|  | Others | 8 (4.3) | 17 (7.3) |  |  |
| Did you receive tetanus toxoid vaccination? | Yes | 556 (91.7) | 333 (75.9) | 50.42 | <0.001* |
|  | No | 50 (8.3) | 106 (24.1) |  |  |
| How many doses did you receive? § | 1 dose | 92 (16.5) | 117 (35.1) | 48.91 | <0.001* |
|  | 2 doses | 391 (70.3) | 191 (57.4) |  |  |
|  | ≥3 doses | 73 (13.1) | 25 (7.5) |  |  |
| Did you receive IPTp-SP during your last pregnancy? | Yes | 505 (83.3) | 284 (64.7) | 48.17 | <0.001* |
|  | No | 101 (16.7) | 155 (35.3) |  |  |
| How many doses of IPTp-SP did you receive? ¶ | 1 dose | 108 (21.4) | 101 (35.6) | 32.47 | <0.001* |
|  | 2 doses | 274 (54.3) | 144 (50.7) |  |  |
|  | ≥3 doses | 123 (24.4) | 39 (13.7) |  |  |
| Did you take folic acid during pregnancy? | Yes | 570 (94.1) | 354 (80.6) | 45.79 | <0.001* |
|  | No | 36 (5.9) | 85 (19.4) |  |  |
| Did you take iron/calcium supplements? | Yes | 548 (90.4) | 309 (70.4) | 66.81 | <0.001* |
|  | No | 58 (9.6) | 130 (29.6) |  |  |
| Did you use any family planning method before pregnancy? | Yes | 390 (64.4) | 185 (42.1) | 51.62 | <0.001* |
|  | No | 216 (35.6) | 254 (57.9) |  |  |
| Did you consume additional meals during pregnancy? | Yes | 487 (80.4) | 266 (60.6) | 50.11 | <0.001* |
|  | No | 119 (19.6) | 173 (39.4) |  |  |

*Statistically significant at p < 0.05. † Percentages for ANC timing and number of ANC visits were calculated using only respondents who attended ANC during their last pregnancy (Urban n = 575; Rural n = 363); ‡ Percentages for danger signs experienced during pregnancy were calculated using only respondents who experienced danger signs during pregnancy (Urban n = 186; Rural n = 232); § Percentages for tetanus toxoid doses received were calculated using only respondents who received tetanus toxoid vaccination during pregnancy (Urban n = 556; Rural n = 333); ¶ Percentages for IPTp-SP doses received were calculated using only respondents who received IPTp-SP during pregnancy (Urban n = 505; Rural n = 284).

**Table S5. Association Between Sociodemographic Characteristics and Good Maternal Healthcare Knowledge Among Respondents (N = 1,045)**

| **Variable** | **Category** | **Good Knowledge**  **n (%)** | **Poor/Moderate Knowledge**  **n (%)** | **χ²** | **p-value** |
| --- | --- | --- | --- | --- | --- |
| Place of residence | Urban | 402 (66.3) | 204 (33.7) | 92.47 | <0.001* |
|  | Rural | 112 (25.5) | 327 (74.5) |  |  |
| Age group (years) | <30 years | 258 (44.3) | 324 (55.7) | 6.52 | 0.011* |
|  | ≥30 years | 256 (55.3) | 207 (44.7) |  |  |
| Marital status | Married | 448 (52.7) | 402 (47.3) | 3.24 | 0.072 |
|  | Others | 66 (33.9) | 129 (66.1) |  |  |
| Highest level of education | None/Primary | 83 (27.5) | 219 (72.5) | 118.61 | <0.001* |
|  | Secondary/Tertiary | 431 (58.0) | 312 (42.0) |  |  |
| Employment status | Employed/Self-employed | 372 (60.1) | 247 (39.9) | 41.37 | <0.001* |
|  | Unemployed | 142 (33.3) | 284 (66.7) |  |  |
| Monthly household income | < ₦50,000 | 157 (34.7) | 296 (65.3) | 73.18 | <0.001* |
|  | ≥ ₦50,000 | 357 (60.2) | 235 (39.8) |  |  |
| ANC attendance during last pregnancy | Yes | 496 (52.9) | 442 (47.1) | 31.44 | <0.001* |
|  | No | 18 (16.8) | 89 (83.2) |  |  |
| Are maternal healthcare services affordable? | Yes | 364 (62.2) | 221 (37.8) | 58.91 | <0.001* |
|  | No | 150 (32.6) | 310 (67.4) |  |  |
| Are healthcare workers respectful during ANC/PNC visits? | Yes | 448 (57.1) | 337 (42.9) | 36.74 | <0.001* |
|  | No | 66 (25.4) | 194 (74.6) |  |  |
| Do you feel safe attending health facilities? | Yes | 469 (55.2) | 380 (44.8) | 28.33 | <0.001* |
|  | No | 45 (23.0) | 151 (77.0) |  |  |

*Statistically significant at p < 0.05.

**Table S6. Association Between Sociodemographic Characteristics and Positive Maternal Healthcare Attitude Among Respondents (N = 1,045)**

| **Variable** | **Category** | **Positive Attitude**  **n (%)** | **Negative Attitude**  **n (%)** | **χ²** | **p-value** |
| --- | --- | --- | --- | --- | --- |
| Place of residence | Urban | 528 (87.1) | 78 (12.9) | 156.42 | <0.001* |
|  | Rural | 228 (51.9) | 211 (48.1) |  |  |
| Age group (years) | <30 years | 387 (66.5) | 195 (33.5) | 8.63 | 0.003* |
|  | ≥30 years | 369 (79.7) | 94 (20.3) |  |  |
| Marital status | Married | 647 (76.1) | 203 (23.9) | 4.41 | 0.036* |
|  | Others | 109 (55.9) | 86 (44.1) |  |  |
| Highest level of education | None/Primary | 145 (48.0) | 157 (52.0) | 127.85 | <0.001* |
|  | Secondary/Tertiary | 611 (82.2) | 132 (17.8) |  |  |
| Employment status | Employed/Self-employed | 507 (81.9) | 112 (18.1) | 74.28 | <0.001* |
|  | Unemployed | 249 (58.5) | 177 (41.5) |  |  |
| Monthly household income | < ₦50,000 | 253 (55.8) | 200 (44.2) | 86.67 | <0.001* |
|  | ≥ ₦50,000 | 503 (84.9) | 89 (15.1) |  |  |
| Good maternal healthcare knowledge | Yes | 468 (91.1) | 46 (8.9) | 211.48 | <0.001* |
|  | No (Poor/Moderate) | 288 (54.2) | 243 (45.8) |  |  |
| Are maternal healthcare services affordable? | Yes | 487 (83.2) | 98 (16.8) | 67.31 | <0.001* |
|  | No | 269 (58.5) | 191 (41.5) |  |  |
| Are healthcare workers respectful during ANC/PNC visits? | Yes | 641 (81.7) | 144 (18.3) | 93.74 | <0.001* |
|  | No | 115 (44.2) | 145 (55.8) |  |  |
| Do you feel safe attending health facilities? | Yes | 675 (79.5) | 174 (20.5) | 76.42 | <0.001* |
|  | No | 81 (41.3) | 115 (58.7) |  |  |

*Statistically significant at p < 0.05.

**Table S7. Association Between Sociodemographic Characteristics and Good Maternal Healthcare Practice Among Respondents (N = 1,045)**

| **Variable** | **Category** | **Good Practice**  **n (%)** | **Poor/Fair Practice**  **n (%)** | **χ²** | **p-value** |
| --- | --- | --- | --- | --- | --- |
| Place of residence | Urban | 350 (57.8) | 256 (42.2) | 118.76 | <0.001* |
|  | Rural | 105 (23.9) | 334 (76.1) |  |  |
| Age group (years) | <30 years | 215 (36.9) | 367 (63.1) | 17.42 | <0.001* |
|  | ≥30 years | 240 (51.8) | 223 (48.2) |  |  |
| Marital status | Married | 398 (46.8) | 452 (53.2) | 5.77 | 0.016* |
|  | Others | 57 (29.2) | 138 (70.8) |  |  |
| Highest level of education | None/Primary | 73 (24.2) | 229 (75.8) | 96.31 | <0.001* |
|  | Secondary/Tertiary | 382 (51.4) | 361 (48.6) |  |  |
| Employment status | Employed/Self-employed | 352 (56.9) | 267 (43.1) | 86.74 | <0.001* |
|  | Unemployed | 103 (24.2) | 323 (75.8) |  |  |
| Monthly household income | < ₦50,000 | 131 (28.9) | 322 (71.1) | 99.06 | <0.001* |
|  | ≥ ₦50,000 | 324 (54.7) | 268 (45.3) |  |  |
| Good maternal healthcare knowledge | Yes | 359 (69.8) | 155 (30.2) | 233.57 | <0.001* |
|  | No (Poor/Moderate) | 96 (18.1) | 435 (81.9) |  |  |
| Positive maternal healthcare attitude | Yes | 404 (53.4) | 352 (46.6) | 157.34 | <0.001* |
|  | No | 51 (17.6) | 238 (82.4) |  |  |
| Are maternal healthcare services affordable? | Yes | 345 (59.0) | 240 (41.0) | 91.82 | <0.001* |
|  | No | 110 (23.9) | 350 (76.1) |  |  |
| Are healthcare workers respectful during ANC/PNC visits? | Yes | 404 (51.5) | 381 (48.5) | 68.57 | <0.001* |
|  | No | 51 (19.6) | 209 (80.4) |  |  |
| Do you feel safe attending health facilities? | Yes | 415 (48.9) | 434 (51.1) | 46.83 | <0.001* |
|  | No | 40 (20.4) | 156 (79.6) |  |  |

*Statistically significant at p < 0.05.
